# Ovarian cancer surgery in Germany: An analysis of the nationwide hospital file 2005–2015

**DOI:** 10.1177/17455065221075903

**Published:** 2022-02-04

**Authors:** Pietro Trocchi, Pawel Mach, Karl Rainer Kimmig, Andreas Stang

**Affiliations:** 1 Institute of Medical Informatics, Biometry and Epidemiology (IMIBE), University Hospital of Essen, Essen, Germany; 2 Department of Obstetrics and Gynecology, University Hospital of Essen, Essen, Germany; 3 Department of Epidemiology, School of Public Health, Boston University, Boston, MA, USA

**Keywords:** Germany, hospitalizations, ovarian cancer, population-based, surgical treatment

## Abstract

**Objectives:**

Nationwide hospitalization data on the surgical management of ovarian cancer are scant. We assessed type of surgery, surgical approach, length of stay, surgery-related complications and in-hospital mortality among women with ovarian cancer in Germany. We analyzed nationwide hospitalization file of 2005 through 2015 including 77,589 ovarian cancer-related hospitalizations associated with ovarian surgery.

**Methods:**

We calculated the relative frequency of the surgical approaches by type of surgery and calendar time. We used log-binomial regression models to estimate relative risk of in-hospital mortality (including 95% confidence intervals) according to complications. About 63% of the hospitalizations included an additional hysterectomy besides ovariectomy.

**Results:**

About 85% of the surgeries were performed by laparotomy. However, from 2005–2006 through 2013–2015, the proportion of laparoscopic ovariectomies (±salpingectomy) increased from 14% to 35%. The in-hospital mortality risks for laparotomic and laparoscopic surgery were 2.9% and 0.4%, respectively. Adjusted mortality risk ratios varied from 1.35 (95% confidence interval = 0.94–1.94) for bleedings requiring blood transfusion to 3.65 (95% confidence interval = 3.31–4.03) for postoperative infections.

**Conclusion:**

We observed a tendency away from laparotomy toward laparoscopy for ovariectomies (±salpingectomy) over time. Compared with laparotomy, laparoscopy was associated with lower risk of complications and death. All complications studied were associated with higher in-hospital mortality risk.

## Introduction

Ovarian cancer is the leading cause of mortality from gynecological malignancies.^1,2^ In Europe, ovarian cancer accounts for 4.1% of all newly diagnosed female malignancies (after excluding non-melanoma skin cancer) and 5.5% of all cancer deaths among women.^1^ In Germany, the estimated number of newly diagnosed cases of ovarian cancer and ovarian cancer deaths in 2014 is 7,250 and 5,350, respectively.^3^

According to the European guidelines, the standard management of ovarian cancer includes the complete tumor cytoreduction followed by platinum- and taxane-based chemotherapy for advanced stages.^4^ Since up to 30% of patients with early-stage ovarian cancer have occult lymph node or peritoneal metastases,^5,6^ the German guidelines recommend for these patients performing an adequate laparotomic operative staging, including total hysterectomy, adnex extirpation, total omentectomy, appendectomy and para-aortic and pelvic lymphadenectomy.^7^

Typical complications after surgery for ovarian cancer include hemorrhages and acute posthemorrhagic anemia, blood transfusions, postoperative infections (including peritonitis), organ perforations and prolonged in-hospital stay. Several studies have assessed the risk of operative morbidity and perioperative mortality among ovarian cancer patients by comparing oncological outcomes of laparoscopic and laparotomic approaches for the treatment of ovarian cancer. However, to the best of our knowledge, no study provided population-based information on surgical treatment of ovarian cancer based on nationwide in-hospital statistics.

Based on nationwide hospitalization data of the years 2005 up to 2015, the goal of this study was to describe the surgical management of ovarian cancer in Germany and to study in-hospital mortality, surgery-related complications and length of hospital stay in relation to the type of surgery and the surgical approach among patients with ovarian cancer.

## Methods

According to the hospital financing law (Kranken-hausentgeltsgesetz, KHEntG), general hospitals in Germany annually transfer their individual hospitalization data to a DRG (diagnosis-related group) data center (Institute for the Hospital Remuneration System, InEK). The DRG data center undertakes a plausibility check of the data and forwards anonymised data to the Federal Bureau of Statistics. Based on confidentiality regulations (Bundesstatistikgesetz, BStatG), individual hospitalization data are available for scientific use. DRG-based hospitalization data include 1 primary diagnosis and up to 89 secondary diagnoses coded by ICD-10 (International Classification of Diseases, 10th edition). Up to 100 medical procedures coded according to a national classification of operations and procedures (OPS) can be documented for each hospitalization case.^8^ For women who undergo ovarian surgery because of ovarian cancer, the diagnosis of ovarian cancer is coded as main diagnosis as this diagnosis is the diagnosis that led to the hospitalization assessed at the end of the hospitalization. DRG hospitalization data do not contain ICD-O codes that would enable the distinction of histological subtypes, grading information or TNM staging information.

We performed a retrospective observational study based on secondary data. Principles of the analysis of this hospitalization file have been described previously. In brief, we identified all hospitalizations of women from 2005 through 2015 with main diagnosis of primary malignant ovarian cancer (ICD-10: C56) that included surgery of the ovary (OPS-Codes: 5-652 to 5-685). Hospitalizations with ovarian surgeries were subdivided into those including hysterectomy (abbreviated HYS, OPS: 5-682, 5-683, 5-685) and those not including hysterectomy, that is, ovariectomy ± salpingectomy only (abbreviated SOV, OPS: 5-652, 5-653). As several codes for surgical procedures of interest may have been assigned, we checked first whether a code for HYS was used and, only if not, whether a code for SOV was used. Surgical procedures were classified by surgical approach by using OPS codes as open abdominal (laparotomic), laparoscopic, vaginal, conversion to open abdominal and other surgical approaches. For each hospitalization, we extracted information on calendar year, age of patient at hospital admission, length of hospital stay in days, lymphadenectomies, surgery-related complications (bleeding or acute posthemorrhagic anemia (ICD-10: D62, K66.1, R58, T81.0), bleeding requiring blood transfusion (OPS: 8-800.0, 8-800.1), postoperative infection (ICD-10: K65, T81.4) and organ perforation (ICD-10: T81.2)) and in-hospital mortality. We derived information on comorbidities according to the Charlson Comorbidity Index using a coding algorithm for defining comorbidities in ICD-10 administrative databases.^9^

### Statistical methods

We analyzed nationwide DRG-based in-hospital statistics (DRG statistics) that covers a population of 82 million people in 2015 (thereof 42 million women). We therefore did not undertake sample size calculation. The unit of analysis was the hospital admission of women with main diagnosis of ovarian cancer and ovarian surgery. We excluded few hospitalizations (<0.5%) for the following reasons: missing sex of the patient, place of residence outside Germany, homeless people and unknown places of residence, resulting in a final data set of 77,589 hospitalization cases. We estimated overall and age-specific nationwide surgery rates (per 100,000 person years) by dividing the number of hospitalizations by the midyear population of women. Population data were provided by the Federal Bureau of Statistics. In addition, we calculated relative frequencies of the surgical approaches according to the type of surgery (HYS and SOV). Surgery rates and percentages were estimated according to the following calendar times: 2005–2006, 2007–2009, 2010–2012 and 2013–2015. Hospitalizations with missing calendar time (N = 183, 0.2%) were excluded from calendar-time stratified analyses. We used log-binomial regression models to estimate age- and comorbidities-adjusted relative risks (RR) and corresponding 95% confidence intervals (95% CIs) of in-hospital mortality in relation to surgery-related complications. All analyses were performed with SAS® (SAS Inc., Cary, NC, USA), Version 9.4.

### Ethics

In compliance with the German confidentiality regulations, the Federal Bureau of Statistics makes individual hospitalization data available for scientific uses without ethical review (§16, (6), (7)). Individual DRG hospitalization data are not publicly available. We sought permission in order to access the data. Because the data are anonymized, meaning that patients cannot be re-identified, informed consent was not required (Professional regulation for North Rhine physicians, §15 Research (1), (3)).

## Results

In Germany, from 2005 through 2015, 77,589 hospitalizations were associated with main diagnosis of ovarian cancer and ovarian surgery. The median length of hospital stay was 14 days. About 63% of the hospitalizations included a HYS. About 85% of the ovarian surgeries were performed by open abdominal surgical approach. The most frequent surgery-related complication was bleeding or acute posthemorrhagic anemia (37%), and the in-hospital mortality risk was 2.6% (Table 1). The relative frequency of hospitalizations that included lymphadenectomy increased from 31% in the years 2005–2006 to 40% in the years 2013–2015 (Supplementary Table S1).

**Table 1.** Characteristics of patients undergoing surgical treatment of malignant cancer of ovary (ICD-10: C56) in Germany 2005–2015.

Hospitalizations: N	77,589
Age (years) at hospitalization: %	
<40	6.9
40–49	14.6
50–59	21.9
60–69	24.7
70–79	23.7
⩾80	8.2
Length of hospital stay (days): Median (P10, P90)	14 (6, 29)
In-hospital deaths: %	2.6
Charlson comorbidity index: Median (P10, P90)	3 (2, 6)
Type of surgery: %	
Hysterectomy	62.7
(Salpingo-)Ovariectomy	37.3
Lymphadenectomy: %	37.2
Surgical approach: %	
Open abdominal	85.4
Laparoscopic	10.8
Vaginal	0.7
Conversion to open abdominal	1.6
Other	1.5
Complications: %	
Bleeding	37.2
Transfusion	0.9
Infection	6.3
Perforation	4.4

P10 and P90: 10th and 90th percentile; Hysterectomy: ovariectomy plus radical, total or subtotal hysterectomy; (Salpingo-)Ovariectomy: only ovariectomy ± salpingectomy.

Overall, the rates of hospitalizations with HYS were almost constant over time from 2005–2006 to 2010–2012, while in 2013–2015 there was a slight decrease of the rate. The rate of hospitalizations that included SOV increased monotonically from 5.6 to 6.8 per 100,000 person years (rate difference = 1.2, 95% CI = 1.0–1.4 per 100,000 person years). In particular, we observed an increase in these rates among women aged <60 years (Table 2).

**Table 2.** Overall and age-specific surgery rates (per 100,000 person years) with main diagnosis of malignant cancer of ovary (ICD-10: C56) by type of operation and calendar time in Germany 2005–2015.

	Overall		Age group											
			0–39		40–49		50–59		60–69		70–79		80+	
	Rate	SE	Rate	SE	Rate	SE	Rate	SE	Rate	SE	Rate	SE	Rate	SE
Hysterectomy														
2005–2006	10.7	0.1	1.3	0.1	11.7	0.3	19.0	0.4	24.5	0.5	24.2	0.6	11.4	0.5
2007–2009	10.8	0.1	1.3	0.0	11.6	0.2	18.5	0.3	23.4	0.4	24.1	0.4	11.8	0.4
2010–2012	10.7	0.1	1.2	0.0	11.1	0.2	18.4	0.3	23.0	0.4	23.3	0.4	10.7	0.4
2013–2015	10.2	0.1	1.1	0.0	11.2	0.2	18.0	0.3	20.8	0.4	20.5	0.4	10.0	0.3
(Salpingo-)Ovariectomy														
2005–2006	5.6	0.1	1.2	0.1	3.2	0.2	7.3	0.3	13.8	0.4	14.8	0.4	9.9	0.4
2007–2009	6.2	0.1	1.5	0.1	4.2	0.1	7.8	0.2	13.6	0.3	15.7	0.4	10.3	0.4
2010–2012	6.3	0.1	1.6	0.1	4.5	0.2	7.8	0.2	13.2	0.3	16.0	0.3	9.1	0.3
2013–2015	6.8	0.1	1.9	0.1	5.4	0.2	9.2	0.2	12.5	0.3	16.4	0.3	8.8	0.3

SE: standard error; Hysterectomy: ovariectomy plus radical, total or subtotal hysterectomy; (Salpingo-)Ovariectomy: only ovariectomy ± salpingectomy.

From 2005–2006 through 2013–2015, the proportion of hospitalizations including HYS decreased from 66% to 60% and, conversely, the proportion of hospitalizations including SOV increased from 34% to 40%. The vast majority of HYS were performed by open abdominal surgical approach. However, the proportion of these surgeries decreased over time from 97% to 92%, while the proportion of laparoscopic HYS increased from 0.4% to 4%. Similarly, the proportion of laparotomic SOV decreased from 82% to 61%, while the proportion of laparoscopic SOV increased from 14% to 35% (Table 3).

**Table 3.** Hospitalizations with main diagnosis of malignant cancer of ovary (ICD-10: C56) and ovarian surgery by type of operation, surgical approach and calendar time in Germany 2005–2015 (N = 77,406).

	Calendar year				
	2005–2006	2007–2009	2010–2012	2013–2015	Overall
Hysterectomy: N (%)	9,025 (65.7)	13,531 (63.6)	13,356 (62.8)	12,644 (59.9)	48,556 (62.7)
Open abdominal: %	96.7	95.5	94.7	92.1	94.6
Conversion to open abdominal: %	0.5	0.7	0.6	0.9	0.7
Laparoscopic: %	0.4	1.0	2.0	4.0	2.0
Vaginal: %	0.7	1.0	1.2	1.4	1.1
Other: %	1.6	1.7	1.4	1.6	1.6
(Salpingo-)Ovariectomy: N (%)	4,712 (34.3)	7,756 (36.4)	7,918 (37.2)	8,464 (40.1)	28,850 (37.3)
Open abdominal: %	81.6	74.8	67.5	61.2	69.9
Conversion to open abdominal: %	2.6	2.9	3.4	3.2	3.1
Laparoscopic: %	13.9	20.7	28.1	34.9	25.8
Vaginal: %	0.1	0.1	0.2	0.0	0.1
Other: %	1.8	1.5	0.8	0.7	1.1

Hysterectomy: ovariectomy plus radical, total or subtotal hysterectomy; (Salpingo-)Ovariectomy: only ovariectomy ± salpingectomy.

**Figure 1.**
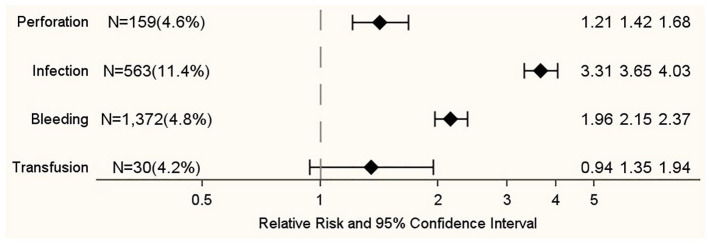
Association between complications and in-hospital death during hospitalizations with main diagnosis of malignant cancer of ovary (ICD-10: C56) and ovarian surgery in Germany 2005–2015. Relative risks are adjusted for age and Charlson comorbidity index. For each complication, the reference group consists of hospitalizations without the corresponding complication.

In comparison to hospitalizations that included HYS, hospitalizations with SOV were associated with 3 days shorter hospital stay, lower risk of all surgery-related complications and a slightly higher relative frequency of in-hospital death (3.0% vs 2.4%). Compared to laparoscopic surgeries, open abdominal surgeries were associated with prolonged hospital stays (15 vs 4 days), higher risk of all surgical complications and higher in-hospital mortality risk (2.9% vs 0.4%). In particular, the risk of bleeding for laparotomic surgeries was more than 10 times the risk of bleeding for laparoscopic surgeries (42% vs 4%) (Table 4). All surgery-related complications were associated with increased adjusted RR of in-hospital mortality, varying from 1.35 (95% CI = 0.94–1.94) for bleedings requiring blood transfusion to 3.65 (95% CI = 3.31–4.03) for postoperative infections (Figure 1).

**Table 4.** Surgery-related complications, in-hospital death and length of in-hospital stay during hospitalizations with main diagnosis of malignant cancer of ovary (ICD-10: C56) and ovarian surgery by type of operation and surgical approach in Germany 2005–2015.

	LOS (days): Median (P10, P90)	Complication: %				In-hospital death: %
		Transfusion	Bleeding	Infection	Perforation	
Type of operation						
Hysterectomy	15 (9, 30)	1.0	43.5	6.8	4.8	2.4
(Salpingo-)Ovariectomy	12 (3, 28)	0.7	26.5	5.5	3.8	3.0
Surgical approach						
Open abdominal	15 (9, 30)	1.0	41.8	6.9	4.8	2.9
Laparoscopic	4 (2, 13)	0.1	4.0	1.6	1.2	0.4
Vaginal	7 (4, 18)	0	9.9	2.5	1.8	0.5
Conversion to open abdominal	10 (5, 22)	0.5	19.9	5.5	4.7	1.4
Other	16 (9, 35)	1.3	45.3	12.1	5.6	5.9

LOS: length of hospital stay; P10 and P90: 10th and 90th percentile; Hysterectomy: ovariectomy plus radical, total or subtotal hysterectomy; (Salpingo-)Ovariectomy: only ovariectomy ± salpingectomy.

## Discussion

Overall, 63% of the hospitalizations with a surgical treatment of the ovary in Germany included HYS. From 2005 to 2015, this proportion decreased over time and was offset by an increasing proportion of hospitalizations that included SOV. Although the large majority of the ovarian surgeries were performed by laparotomy, the proportion of laparoscopic surgeries increased considerably over time, especially among hospitalizations including SOV. Compared to hospitalizations including SOV, the risk of surgery-related complications was higher for hospitalizations that included HYS. Laparotomic surgery was associated with a considerably higher risk of all studied surgery-related complications and in-hospital mortality than laparoscopic surgery. All studied complications were associated with an increased risk of in-hospital mortality.

Although minimally invasive surgical techniques have improved over the last years, physicians continue to debate the use of laparoscopic surgery for ovarian cancer. Because of the lack of randomized controlled trials, a recent review of the Cochrane collaboration suggests that there is insufficient evidence to quantify risks and benefits of the laparoscopic approach.^10^ We found that the proportion of laparotomic SOV decreased over time from 82% to 61%, while the proportion of laparoscopic SOV increased from 14% to 35%. Similarly, the proportion of HYS performed in Germany by open abdominal surgical approach decreased from 97% to 92%, while the proportion of HYS performed laparoscopically increased from 0.4% to 4%. These results are consistent with those of other studies reporting that the indication for minimally invasive surgery in patients with ovarian cancer is increasing.^11^ However, our findings should be interpreted with caution because DRG statistics do not include information on important parameters as tumor stage, tumor histology, hospital- or surgeon-related factors and patients’ preferences. We can only speculate that advances in technology and surgical skills over time could have contributed to an increasing relative frequency of the use of laparoscopic approach for ovarian cancer in Germany during the study period. Finally, we observed a slight increase in rates of hospitalizations with SOV in the younger age groups, which could reflect a growing effort over time to consider fertility conservation an important concern in the management of pre-menopausal women with ovarian cancer.

Several studies assessed length of hospital stay and risk of surgery-related complications in patients with ovarian cancer by comparing laparoscopy with laparotomy. Unfortunately, these studies used different definitions of operative complications, thus limiting the comparability of their results with our findings. A number of these studies reported that intraoperative complication rates among patients with early-staged ovarian cancer were similar regardless of the surgical approach.^12–15^ In contrast, a recent systematic review from Italy found that patients with early-stage ovarian cancer undergoing laparoscopy experienced lower postoperative complication rates than patients undergoing laparotomy.^16^ Another recent hospital-based study reported lower intraoperative complication rates and considerably lower rates of intraoperative transfusions (9.4% vs 55.9%) for laparoscopy when compared with laparotomy among patients with advanced ovarian cancer.^17^ Laparoscopy has been associated in several studies with shorter length of hospital stay when compared with laparotomy.^12–15,18–20^ Our analyses of in-hospital statistics in Germany included women with ovarian cancer at different stages and showed that ovarian surgeries performed laparoscopically were associated with considerably shorter median hospital stay (4 vs 15 days), lower risk of surgery-related complications and considerably lower risk of in-hospital mortality (0.4% vs 2.9%) in comparison to ovarian surgeries performed by open abdominal surgical approach. These findings may be partly explained by confounding by indication (tumor histology, tumor stage, and grade) that we could not take into account in our analyses. However, our statistics showed that hospitalizations including SOV were associated with lower complication rates if compared with those including HYS. In addition, SOV accounted for the large majority of the laparoscopic surgeries (N = 7,444 out of 8,402, 89%), while HYS accounted for almost 70% of the surgeries performed by abdominal open surgical approach (N = 45,949 out of 66,123). The lower risk of complications for laparoscopies than for laparotomies estimated in our study is therefore mainly due to the high proportion of laparoscopic SOV that are usually performed for diagnostic purposes and not for curative purposes.

In the current study, the in-hospital mortality was 2.6% and all surgery-related complications were associated with an increased adjusted in-hospital mortality risk, with the highest risk among women with postoperative infections when compared with women without this complication. These findings are in line with those provided by a systematic review of Gerestein et al.^21^ who reported a perioperative mortality among patients with advanced ovarian cancer varying from 2.5% to 4.8%. In addition, according to this review, sepsis is the second most common cause of mortality (21% of all cases) after pulmonary embolism. However, it should be noted that, unlike our study, mortality was defined as death from any cause within 30 days of operation in almost all the reports included in this review.

The use of administrative data in clinical research has some important limitations. First, several factors beyond patient age, comorbidities and type of surgery are related to peri- and postoperative morbidity and mortality like tumor stage, tumor histology and grade. Because of missing information on these factors, we could not stratify the analyses based on disease’s severity and tumor entity. Second, as administrative hospitalization data lack information on grade of complications, neither the Clavien classification of surgical complications^22^ nor the Memorial Sloan-Kettering Cancer Center surgical secondary events grading system^23^ could be applied. Third, owing to the legal anonymization of the data, women who were hospitalized more than once could not be identified. Therefore, we were not able to assess the association between complications and risk of re-operations after discharge. Finally, our results are derived from 2005 to 2015 and management of ovarian cancer may have changed in favor of robotic surgery.

## Conclusion

In conclusion, our analyses of the DRG statistics for the years 2005 up to 2015 provide for the first time nationwide details on the surgical management of ovarian cancer in Germany. The majority of ovarian surgeries were performed by open abdominal approach. However, there was a strong shift over time of SOV performed by this approach toward SOV performed laparoscopically. When compared to surgeries performed by open abdominal approach, laparoscopic surgeries were associated with shorter length of stay, lower risk of surgery-related complications and lower in-hospital mortality risk, which may be due to confounding by indication that we could not address in our study.

## Supplemental Material

sj-docx-1-whe-10.1177_17455065221075903 – Supplemental material for Ovarian cancer surgery in Germany: An analysis of the nationwide hospital file 2005–2015 Click here for additional data file. Supplemental material, sj-docx-1-whe-10.1177_17455065221075903 for Ovarian cancer surgery in Germany: An analysis of the nationwide hospital file 2005–2015 by Pietro Trocchi, Pawel Mach, Karl Rainer Kimmig and Andreas Stang in Women’s Health
