# Supplementary material for: Ovarian cancer surgery in Germany: An analysis of the nationwide hospital file 2005–2015
Source: Womens Health (Lond). 2022 Feb 4;18:17455065221075903. doi: 10.1177/17455065221075903 (PMC8819748; doi:10.1177/17455065221075903)
Supplement: sj-docx-1-whe-10.1177_17455065221075903 – Supplemental material for Ovarian cancer surgery in Germany: An analysis of the nationwide hospital file 2005–2015 [file sj-docx-1-whe-10.1177_17455065221075903.docx]

**Supplementary Table S1.** Lymphadenectomy during hospitalizations with main diagnosis of malignant cancer of ovary (ICD-10: C56) and ovarian surgery by calendar time in Germany 2005-15.

|  | Lymphadenectomy | |
| --- | --- | --- |
|  | N | % |
| Calendar time |  |  |
| 2005-2006 | 4,297 | 31.3 |
| 2007-2009 | 8,109 | 38.1 |
| 2010-2012 | 8,092 | 38.0 |
| 2013-2015 | 8,348 | 39.6 |
